# The Western Medico-Chirurgical Journal

**Published:** 1850-10

**Authors:** 

**Affiliations:** Professor in the Iowa State University; Professor in the Iowa State University


*The Western Medico-Chirurgical Journal.* Edited by J. F. Sanford, M. D., and Samuel. G. Armor, M. D., Professors in the Iowa State University.

The Far West is rapidly growing in population, and the young State of Iowa, already boasts a medical school, and a medical journal devoted to the interests of science.

The two numbers of this new journal, the first and second, which we have received, speak favorably of the abilities of the editors, and we hope they will succeed well in the responsible duties they have undertaken. B.

